# Overexpression of SphK2 contributes to ATRA resistance in colon cancer through rapid degradation of cytoplasmic RXRα by K48/K63-linked polyubiquitination

**DOI:** 10.18632/oncotarget.17174

**Published:** 2017-04-18

**Authors:** Wen-Na Shi, Shu-Xiang Cui, Zhi-Yu Song, Shu-Qing Wang, Shi-Yue Sun, Xin-Feng Yu, Ye Li, Yu-Hang Zhang, Zu-Hua Gao, Xian-Jun Qu

**Affiliations:** ^1^ Department of Pharmacology, School of Basic Medical Sciences, Capital Medical University, Beijing, China; ^2^ Beijing Key Laboratory of Environmental Toxicology, Department of Toxicology and Sanitary Chemistry, School of Public Health, Capital Medical University, Beijing, China; ^3^ Department of Pathology, McGill University, Montreal, Quebec, Canada

**Keywords:** sphingosine kinase 2 (SphK2), retinoid therapy resistance, cytoplasmic RXRα, polyubiquitination

## Abstract

The resistance mechanisms that limit the efficacy of retinoid therapy in cancer are poorly understood. Sphingosine kinase 2 (SphK2) is a highly conserved enzyme that is mainly located in the nucleus and endoplasmic reticulum. Unlike well-studied sphingosine kinase 1 (SphK1) located in the cytosol, little has yet understood the functions of SphK2. Here we show that SphK2 overexpression contributes to the resistance of all-trans retinoic acid (ATRA) therapy in colon cancer through rapid degradation of cytoplasmic retinoid X receptor α (RXRα) by lysine 48 (K48)- and lysine 63 (K63)-based polyubiquitination. Human colonic adenocarcinoma HCT-116 cells transfected with SphK2 (HCT-116^Sphk2^ cells) demonstrate resistance to ATRA therapy as determined by *in vitro* and *in vivo* assays. Sphk2 overexpression increases the ATRA-induced nuclear RXRα export to cytoplasm and then rapidly degrades RXRα through the polyubiquitination pathway. We further show that Sphk2 activates the ubiquitin-proteasome system through the signal mechanisms of (1) K48-linked proteosomal degradation and (2) K63-linked ubiquitin-dependent autophagic degradation. These results provide new insights into the biological functions of Sphk2 and the molecular mechanisms that underlie the Sphk2-mediated resistance to retinoid therapy.

## INTRODUCTION

Retinoid therapy improves the outcomes of many cancers. The biological function of retinoids is achieved through binding with their nuclear DNA-binding receptors retinoic acid receptors (RARs) and retinoid X receptors (RXRs). In response to retinoid therapy, RARs and RXRs act as RXRs-RARs heterodimers that bind to a variety of retinoic acid response elements (RARE) sequences which regulate gene transcription through recruitment of corepressors and coactivators [1, 2]. RXRα (retinoid X receptor-α) predominately functions as a transcription factor with roles in cell development, differentiation, metabolism and death, etc. In nucleus, RXRα dimerizes either with other nuclear receptors to form heterodimers or with itself to form homodimers that bind to specific DNA-response elements in the promoter regions of target genes to release corepressors and recruit coactivators, permitting the multiprotein transcriptional machinery to initiate transcription [3, 4].

Recently, cytoplasmic functions of RXRα have been found that are distinct from its activity as a transcription factor [5]. RXRα translocates from the nucleus to the cytoplasm, resulting in differentiation, survival and apoptosis, etc [6]. The translocation of RXRα from the nucleus to the cytoplasm is highly regulated by its dimerization and ligand binding. The translocation of RXRα/Nur77 heterodimer from the nucleus to the cytoplasm leads to cancer apoptosis [7]. The translocation of RXRα/Nur77 heterodimer between the nucleus and the cytoplasm can be regulated by RXRα ligand [8]. RXRα has thus been considered to have pleiotropic biological actions [9]. However, only one-third of cancer patients respond to retinoid therapy and the resistance mechanisms that limit the efficacy of retinoids in cancer are poorly understood [10].

Sphingosine kinase (SphK) is a highly conserved enzyme that catalyzes the phosphorylation of sphingosine to produce sphingosine 1-phosphate (S1P). Two distinct isoforms of SphKs, SphK1 and SphK2, have been identified. Unlike well-studied SphK1 [11], little has yet been known about the functions of SphK2. SphK2 is present in intracellular compartments, mainly nucleus and mitochondria [12]. Accumulating reports show that high expression of SphK2 is frequently occurs in many cancers [13–15]. However, there has not been any report yet showing an association between SphK2 expression and therapeutic outcomes in patients with the retinoid therapy. Recently, we reported that overexpression of SphK2 correlated to the loss of RARβ and RXRα in colonic cancer cells [16]. Cancer cells transfected with SphK2 antagonize all-trans retinoic acid (ATRA)-induced RARβ promoter activity through acetylated RARβ degradation. SphK2 could also increase the ligand-dependent degradation of RXRα, which in turn counteracts ATRA activation of the RXRα/RARβ heterodimer. In this study, we found that the resistance to ATRA therapy is largely due to overexpression of SphK2, which increases ATRA-induced nuclear RXRα export to the cytoplasm, and then enhances its subsequent degradation through activation of the K48- and K63-based polyubiquitination pathway. Our study highlights the association between SphK2 and abnormal modulation of RXRα/RARβ in cancer cells. Strategies that disrupt the association between SphK2 and RXRα/RARβ could potentially increase the efficacy of retinoids therapy.

## RESULTS

### Sphk2 and RXRα colocalize in the same subcellular compartments in HCT-116^Sphk2^ cells

**Figure 1:**
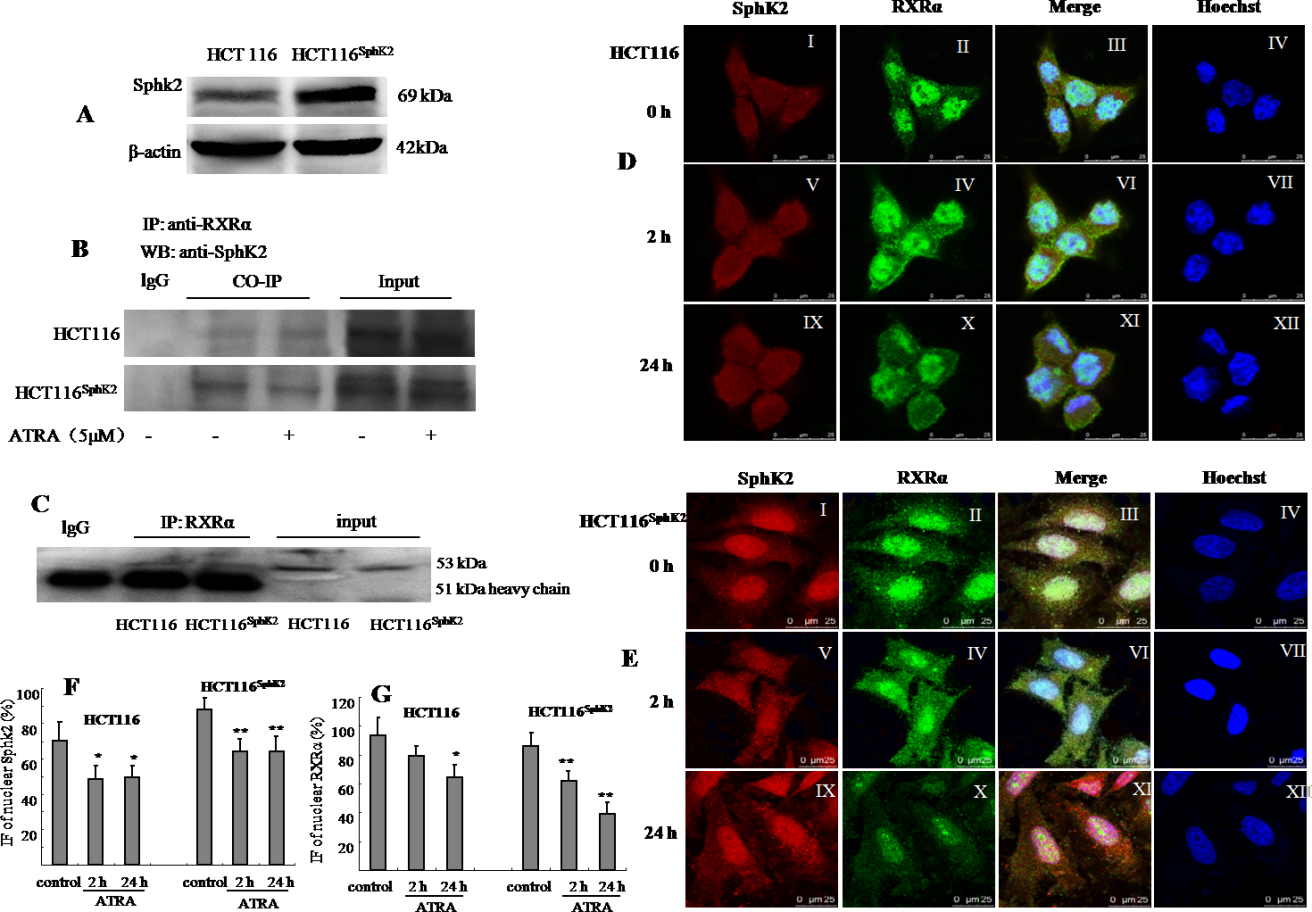
Sphk2 and RXRα are expressed in overlapping subcellular compartments in HCT-116 and HCT-116^Sphk2^ cells **(A)** Western blot of SphK2 in HCT-116 and HCT-116^Sphk2^ cells. **(B)** Western blot of co-immunoprecipitated SphK2 and RXRα in HCT-116 or HCT-116^Sphk2^ treated with or without ATRA for 6 h. HCT-116^Sphk2^ cells showed a strong immunoprecipitate band expressing RXRα contains SphK2. ATRA induced the degradation of RXRα in HCT-116^Sphk2^ cells, showing weaker immunoprecipitate. **(C)** A study to confirm the efficacy of Co-IP in which a primary antibody was assessed for its ability to pull out itself *vs*. normal IgG of the same species which served as the corresponding control. **(D)** and **(E)** Immunofluorescent microsgraphs of the subcellular localization of Sphk2 and RXRα in HCT-116 **(D)** and HCT-116^Sphk2^ **(E)** cells exposed to vehicle (first row), ATRA for 2 h (second row) and ATRA for 24 h (third row). SphK2 staining is shown in the first column (red, I, V, and IX). RXRα staining is shown in the second column (green, II, IV and X). Nuclei are stained with Hoechst (fourth column, IV, VII and XII). A merge of the three channels is shown in the third column. **(D)** Endogenous SphK2 staining is found in the nucleus and cytoplasm in HCT-116 whereas RXRα staining is mainly in nucleus with some residual staining in the cytoplasm. **(E)** Transfected SphK2 mainly resides in the nucleus in HCT-116^Sphk2^ and colocalizes with SphK2 in the absence of ATRA (I). Following ATRA treatment RXRα staining increases in the cytoplasm (2 h, IV) and then disappears in the cytoplasm (24 h, X). **(F)** and **(G)** Immunofluorescence (IF) of nuclear Sphk2 (%) and RXRα (%) were quantitative performed using ImageJ (NIH, http://rsb.info.nih.gov/ij/) and Open View software. Threshold subtraction and measurements of nuclear IF were done in a mask created according to Hoechst staining using NIH Image J. Bars represent means ± S.D. of 3 times. *, p < 0.05 *vs*. vehicle control.

We first examined whether the subcellular distributions of Sphk2 and RXRα overlap in HCT-116 cells and HCT-116^Sphk2^ cells. RXRα localizes in the nucleus. SphK2 mainly resides in the nucleus and shuttles between the cytosol and the nucleus [11, 12]. Western blotting analysis indicated a 2.6-fold increase in SphK2 in HCT-116^Sphk2^ cells as compared to control HCT-116 cells (Figure 1A). Co-immunoprecipitation assays showed that a strong immunoprecipitate band expressing RXRα contains SphK2 in HCT-116^Sphk2^ cells. However, when HCT-116^Sphk2^ cells were exposed to ATRA for 6 h, the immunoprecipitate became weaker, suggesting that ATRA induced the degradation of RXRα in HCT-116^Sphk2^ cells. In contrast, the immunoprecipitate band was not obvious in HCT-116 control cells (Figure 1B). We then analyzed the subcellular distributions of Sphk2 and RXRα and any changes of localization induced by ATRA with immunofluorescence microscopy. SphK2 (red fluorescence) and RXRα (green fluorescence) mainly reside in the nucleus of HCT-116 cells (Figure 1C–I and II). Figure 1D-III shows a merged image if red SphK2 expression and green RXRα expression, indicating significant colocalization in HCT-116^Sphk2^ cells. Immunofluorescence microscopy revealed that nuclear RXRα and Sphk2 were both exported to the cytoplasm in the presence of ATRA. However, cytoplasmic RXRα is rapidly degraded in HCT-116^Sphk2^ cells, as shown by the weaker green fluorescence of RXRα beginning at 2 h post ATRA exposure (Figure 1D–IV). In HCT-116^Sphk2^ cells, the majority of RXRα expression (green fluorescence) disappeared within 24 h ATRA exposure (Figure 1D–X), whereas Sphk2 expression (red fluorescence) remained in the cytoplasm during a 24 h observation (Figure 1D–XI). In contrast, in HCT-116 cells, most of cytoplasmic RXRα remained in cytosol for 24 h post ATRA exposure (Figure 1C–XI). These changes of Sphk2 and RXRα were quantitative analyzed as shown in Figure 1F and 1G. These results indicate that the subcellular distributions of Sphk2 and RXRα in HCT-116^Sphk2^ cells overlap, suggesting a link between Sphk2 and RXRα in expression and function.

### Overexpression of Sphk2 contributes to cancer cell resistance to ATRA therapy

**Figure 2:**
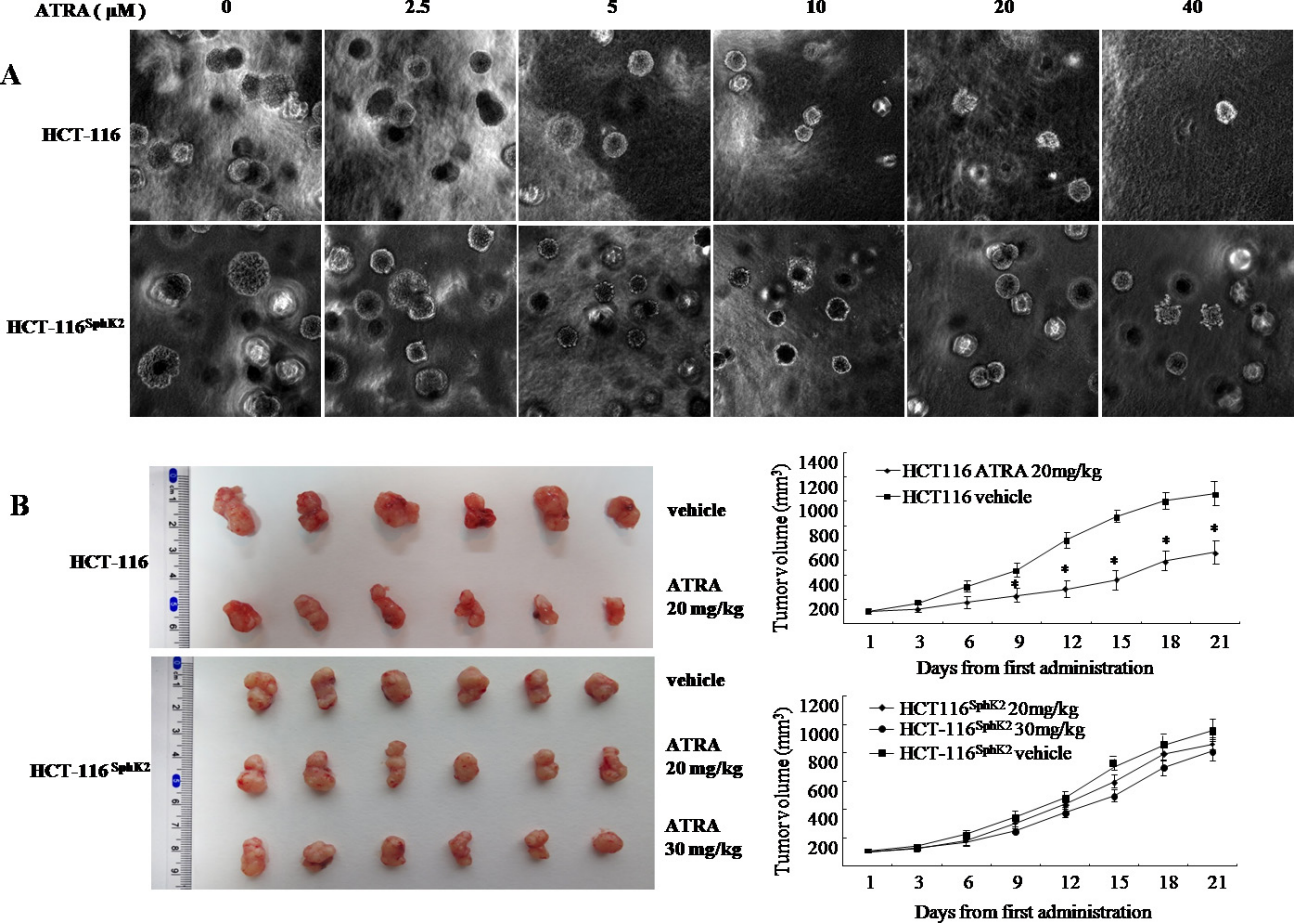
Overexpression of Sphk2 contributes to cancer cell resistance to ATRA therapy **(A)** Soft agar colony assays showing the effect of increasing concentrations of ATRA on HCT-116 and HCT-116^Sphk2^ cell growth. **(B)** Efficacy of ATRA on HCT-116 and HCT-116^Sphk2^ tumor growth in xenografted nude mice. Mice were dosed with vehicle or ATRA at 20 and 30 mg/kg for three consecutive weeks. Tumor volume and body weight were measured every 3 days. Bars represent means ± S.D. of six mice. *, p < 0.05 *vs*. vehicle control.

We compared the sensitivity of HCT-116 and HCT-116^Sphk2^ cells to ATRA therapy as evaluated by *in vitro* and *in vivo* assays. In the soft agar assay, HCT-116 cells demonstrated high sensitivity to various concentrations of ATRA. As shown in Figure 2A, ATRA concentrations of 2.5, 5, 10, 20 and 40 μM significantly inhibited clone formation in HCT-116 cells by 28.9%, 32.5%, 41.8%, 60.7%, and 69.9% (2.5 and 5 μM, p < 0.05; 10 to 40 μM, p < 0.01 *vs*. vehicle control), respectively. In contrast, HCT-116^Sphk2^ cells demonstrated low sensitivity to ATRA therapy. Clone formation in HCT-116^Sphk2^ cells was inhibited by only 13.1%, 18.3%, 22.8%, 26.5% and 34.3% (2.5 and 20 μM, p > 0.05; 40 μM, p < 0.05 *vs*. vehicle control), respectively (Figure 2A). Statistical analysis showed a significant difference between HCT-116 and HCT-116^Sphk2^ cells (p < 0.05).

The reduced response of HCT-116^Sphk2^ cells to ATRA therapy was further demonstrated in nude mice expressing HCT-116^Sphk2^ during three weeks of ATRA therapy. As shown in Figure 2B and Table 1, mice xenografted with HCT-116 cells and dosed with ATRA at 20 mg/kg had smaller tumor volumes than mice dosed with vehicle. ATRA inhibited tumor growth in mice xenografted with HCT-116 by 40.9% (p < 0.05 *vs*. vehicle control). ATRA inhibited cancer growth without a considerable change in food consumption and body weight. In contrast, mice xenografted with HCT-116^Sphk2^ cells and dosed with ATRA at 20 mg/kg showed inhibition of tumor growth by only 10.6% (p > 0.05 *vs*. vehicle control). Increasing the dose of ATRA to 30 mg/kg inhibited tumor growth by 17.3% (p > 0.05 *vs*. vehicle control, Table 1). These results suggest that overexpression of SphK2 contributes to the resistance to ATRA therapy in colon cancer.

**Table 1:** ATRA elicits different responses in HCT-116 and HCT-116^Sphk2^ xenografted nude mice

Cell lines	Treatment (mg/kg)	Number of mice (n)	Body weight (g)^a^(initial/21 days)	Tumor weight^b^(g, mean ± SD)	Tumor growth inhibition (%)
HCT-116	vehicle	6	17.9 ± 1.2/22.4 ± 1.2	1.15 ± 0.39	-
	ATRA 20	6	18.6 ± 1.1/21.2 ± 1.1	0.68 ± 0.21*	40.9
HCT-116^Sphk2^	vehicle	6	18.3 ± 1.3/22.1 ± 0.9	1.04 ± 0.26	-
	ATRA 20	6	18.1 ± 0.8/20.8 ± 1.1	0.93 ± 0.24	10.6
	ATRA 30	6	18.5 ± 0.9/19.5 ± 1.2	0.86 ± 0.27	17.3

Established HCT-116 or HCT-116^Sphk2^ xenografted nude mice were treated with ATRA by gavage once every three days.

^a^Body weight was measured at the indicated time.

^b^Tumors were measured after mice were sacrificed.

*p < 0.05 *vs*. vehicle control.

### Overexpression of Sphk2 enhances the ligand-induced degradation of RARβ and RXRα

**Figure 3:**
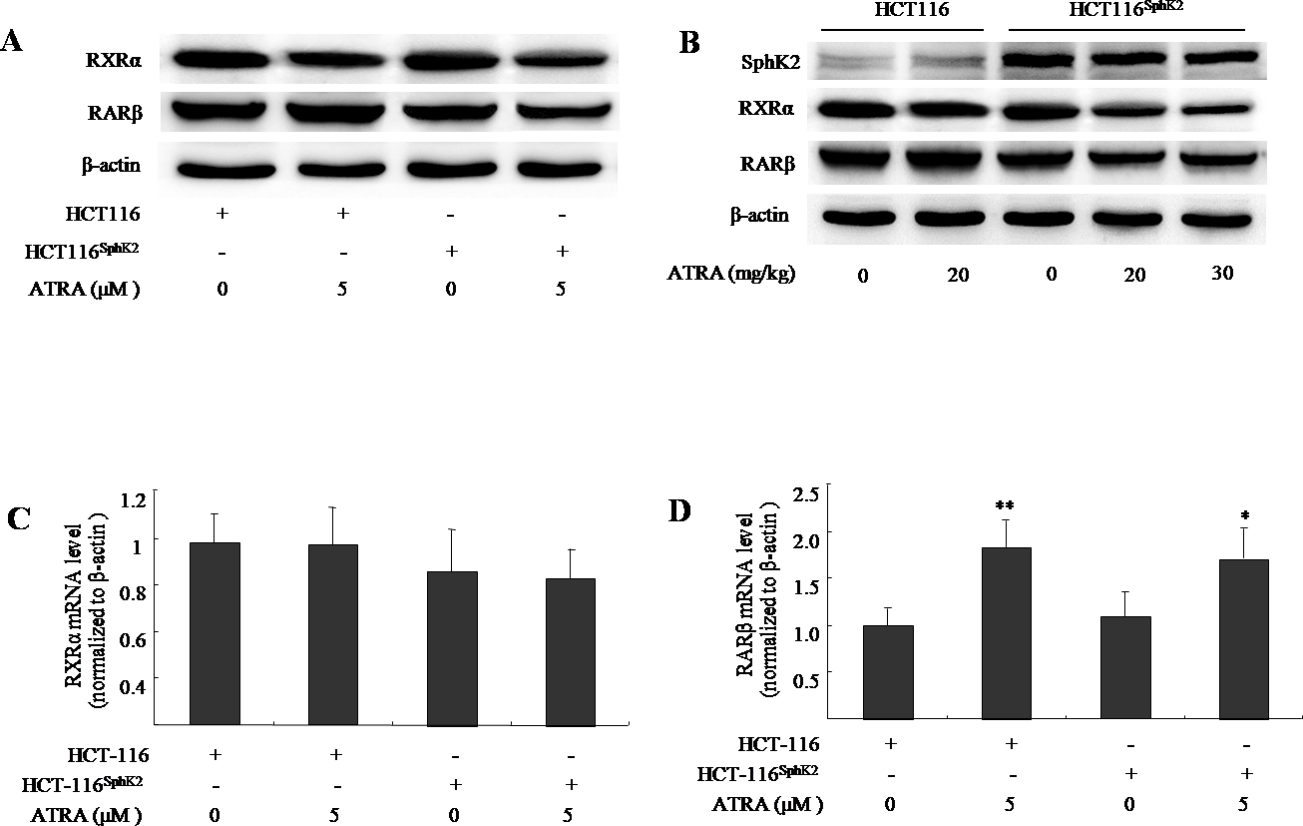
Sphk2 enhances the ATRA-induced degradation of RARβ and RXRα in HCT-116^Sphk2^ cells **(A)** Western blots of cells exposed to vehicle or ATRA (5 μM) for 24 h to analyze the expression of RARβ and RXRα. **(B)** Western blots of xenografts of HCT-116^Sphk2^ or HCT-116 removed from nude mice treated with ATRA for three weeks. Reverse transcriptase and qPCR assays measured RXRα **(C)** and RARβ mRNA. **(D)** HCT-116^Sphk2^ or HCT-116 cells were exposed to ATRA (5 μM) for 24 h and then total RNA was extracted for reverse transcription and qPCR assays to determine the level of RXRα and RARβ mRNAs. The bars indicate means ± S.D (n = 3). *, p < 0.05 *vs*. vehicle control.

We have previously shown that SphK2 and S1P mediate ATRA-induced RARβ degradation through the acetylation degradation pathway [16]. Because RARβ is degraded and RXRα is the obligate partner to RARβ [17], this raises the question of what the fate of RXRα will be in the context of loss of RARβ? Here, we use western blotting analysis and show that the expression of RARβ was activated both in cultured HCT-116 cells exposed to ATRA (5 μM) and in HCT-116 xenografted nude mice dosed with ATRA (20 mg/kg). Conversely, the level of RARβ was significantly reduced in HCT-116^Sphk2^ cells in the presence of ATRA (5 μM) for 24 h and HCT-116^Sphk2^ xenografted nude mice dosed with ATRA (20-30 mg/kg, Figure 3A and 3B). The percentage inhibition on RARβ expression was 65.5% in cultured HCT-116^Sphk2^ and 50.2% and 82.6% in HCT-116^Sphk2^ xenografted mice, respectively. These results confirm that overexpression of Sphk2 enhances ligand-induced RARβ degradation in HCT-116^Sphk2^ cells. Although the expression level of RXRα in both HCT-116 and HCT116^Sphk2^ was decreased upon ATRA treatment, the reduced RXRα in HCT-116^Sphk2^ cells is more serious than in HCT-116 cells (p < 0.05). The expression level of RXRα was reduced by 26.8% in HCT-116 cells (p > 0.05 *vs*. vehicle control) and 64.3% in HCT-116^Sphk2^ cells (p < 0.05 *vs*. vehicle control, Figure 3A) and by 18.2% in HCT116 xenografted mice dosed at 20 mg/kg ATRA (p > 0.05 *vs*. vehicle control, Figure 3B), and by 64.3% and 76.9% in HCT116^Sphk2^ xenografted mice dosed with 20 and 30 mg/kg ATRA, respectively (p > 0.01 *vs*. vehicle control, Figure 3B). Based on these results, we suggest that RXRα might be degraded after ATRA-induced RARβ degradation in HCT-116^Sphk2^ cells. Further, reverse transcriptase and qPCR assays were performed to measure RXRα and RARβ mRNA. No significant change of RXRα mRNA was observed in both HCT-116 and HCT116^Sphk2^ cells exposed to ATRA for 24 h (Figure 3C, p > 0.05 *vs*. vehicle control). RARβ mRNA was significantly increased in HCT-116 (p < 0.01 *vs*. vehicle control) and HCT-116^Sphk2^ cells (Figure 3D, p < 0.05 *vs*. vehicle control). These results indicate that the degradation of RXRα and RARβ did not happen at mRNA level. SphK2 might mediate the ATRA-induced degradation of RARβ and RXRα at the post-translational level.

### SphK2 enhances rapid degradation of RXRα in the cytoplasm through activation of the ubiquitination pathway

**Figure 4:**
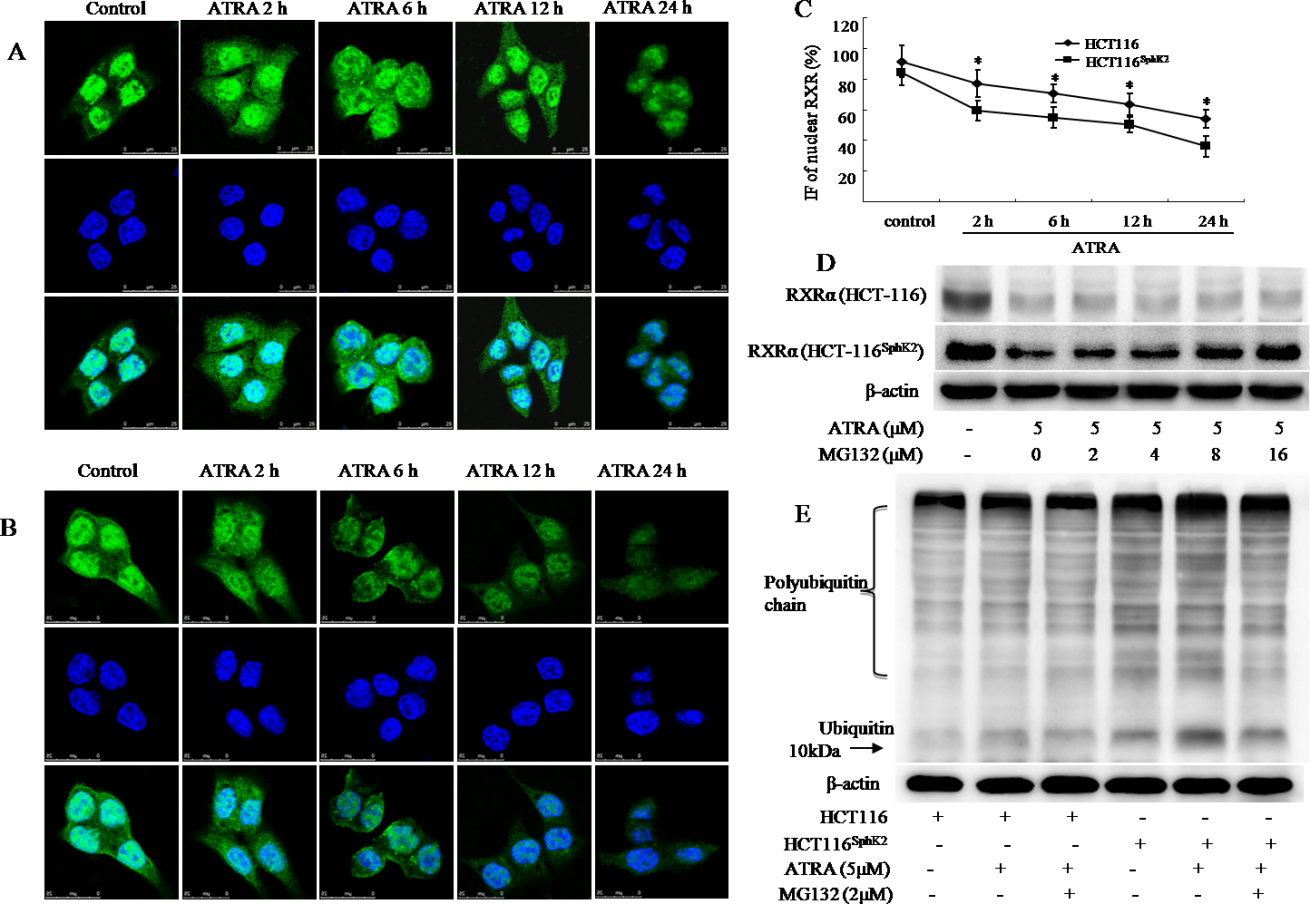
SphK2 enhances the rapid degradation of RXRα in the cytoplasm through the ubiquitination pathway Immunofluorescent micrographs of a time-course of RXRα export to the cytoplasm in HCT-116 **(A)** or HCT-116^Sphk2^ **(B)** cells post ATRA exposure (5 μM). RXRα staining is shown in the first row (green), nuclei are shown in the second row (blue), and merged images are shown in the third row. **(C)** Immunofluorescence (IF) of nuclear RXRα were quantitative analyzed in HCT-116 and HCT-116^Sphk2^ cells exposed to ATRA (5 μM) for 2 to 24 h. The bars indicate means ± S.D (n = 3). p < 0.05 *vs*. HCT-116 cells. **(D)** ATRA-induced degradation of RXRα is blocked by MG132. Western blots of RXRα expression in HCT-116^Sphk2^ or HCT-116 cells exposed to vehicle or ATRA (5 M) with increasing concentrations of MG132 for 24 h. **(E)** Western blots of the ubiquitination of RXRα in HCT-116 or HCT-116^Sphk2^ cells following treatment with ATRA (5 μM) plus MG132 (2 μM) for 24 h. Images of the immunoprecipitates were captured on a FluorChem FC3 image analyzer.

The translocation of RXRα from the nucleus to the cytoplasm represents a unique pathway in the inhibition of cancer growth [6–9]. However, HCT-116^Sphk2^ cells demonstrate that a different mechanism of RXRα translocation and degradation accounts for cancer cell resistance to ATRA therapy. To investigate the mechanism of RXRα translocation and degradation in HCT-116^Sphk2^ cells, we used immunofluorescence microscopy to analyze the subcellular localization of RXRα in the presence of ATRA over time. As shown in Figure 4A and 4B, ATRA could induce the export of nuclear RXRα to the cytoplasm in both HCT-116 and HCT-116^Sphk2^ cells. However, cytoplasmic RXRα was rapidly degraded in HCT-116^Sphk2^ cells in contrast with stable expression in control HCT-116 cells. As shown in Figure 4A, in HCT-116 cells, RXRα mainly resides in the nucleus in the absence of ATRA. A time-course of RXRα expression following ATRA treatment shows that there is high expression of cytoplasmic RXRα at 2 h post ATRA exposure. The majority of RXRα is exported to the cytoplasm at 6 h and it lasts for 24 h when extensive apoptosis occurs (Figure 4A). In HCT-116^Sphk2^ cells, a high level of cytoplasmic RXRα was also observed 2 h post ATRA treatment. But, the fluorescence of RXRα became weaker from 6 h and most RXRα fluorescence had disappeared by 12 h post ATRA treatment. These results indicate that cytoplasmic RXRα is rapidly degraded in HCT-116^Sphk2^ cells upon ATRA treatment (Figure 4B). Vehicle treatment did not have a significant effect on RXRα location and expression in both HCT-116 and HCT-116^Sphk2^ cells. Figure 4C showed a time-dependent reduction of nuclear RXRα determined by quantitative immunofluorescence (IF) analyses in HCT-116 and HCT-116^Sphk2^ cells exposed to ATRA from 2 to 24 h. The level of remained nuclear RXRα was significantly lowered in HCT-116^Sphk2^ cells after 24 h exposure (p < 0.05 *vs*. HCT-116 cells). These results provide compelling evidence that Sphk2 mediates ATRA-induced nuclear RXRα export and then its subsequent degradation in the cytoplasm.

To investigate whether the degradation of RXRα is mediated by the proteasome, we treated HCT-116 and HCT-116^Sphk2^ cells with proteasomal inhibitor MG132, a proteasomal inhibitor [18]. Results show that the degradation of RXRα is significantly blocked by treatment with MG132. Cells were exposed to ATRA with or without MG132 for 24 h. The expression of RXRα was then determined by western blotting analysis. HCT-116^Sphk2^ cells demonstrated a significant decrease in RXRα expression in the presence of ATRA. When HCT-116^Sphk2^ cells were exposed to ATRA (5 μM) plus MG132 with increasing concentrations from 2 to 16 μM, the degradation of RXRα was significantly inhibited. The expression level of RXRα was gradually increased upon treatment with increasing concentrations of MG132 (Figure 4D). MG132 with 8 and 16 μM strongly prevented RXRα degradation (p < 0.01 *vs*. vehicle control). In contrast, MG132-prevented RXRα degradation was not obviously in HCT116 cells (2 and 4 μM, p > 0.05; 8 and 16 μM, p < 0.05 *vs*. vehicle control). These results revealed that ATRA-induced degradation of RXRα in HCT116^SphK2^ cells is more serious than in HCT116 cells. We suggest that RXRα might be degraded through the activation of the proteasomal pathway.

We next examined the expression level of ubiquitin in cell lysates prepared from HCT-116 and HCT-116^Sphk2^ cells. Cell lysates were subjected to anti-ubiquitin antibody to determine the pattern of RXRα degradation. As shown in Figure 4E, HCT-116^Sphk2^ cells had higher expression levels of ubiquitinated proteins than did HCT-116 cells. Strikingly, even higher expression levels of ubiquitinated proteins were observed when HCT-116^Sphk2^ cells were exposed to ATRA (5 μM). MG132 (2 μM) significantly inhibited the formation of ubiquitinated proteins (Figure 4E). These results suggest that SphK2 enhances the ATRA-induced degradation of RXRα in HCT-116^Sphk2^ cells through an ubiquitin pathway.

### SphK2 enhances the conjugation of RXRα with K48- and K63- linked polyubiquitination

**Figure 5:**
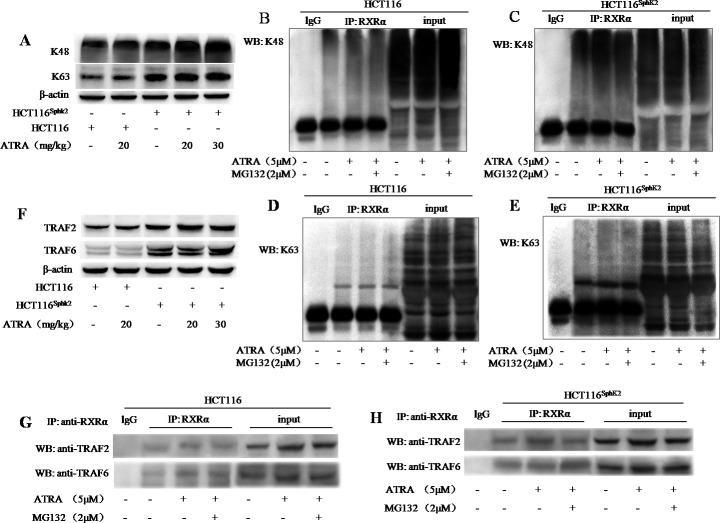
SphK2 enhances the conjugation of RXRα with K48- and K63- based poly-ubiquitination **(A)** Western blots of K48 and K63 in HCT-116 and HCT-116^Sphk2^ xenografts from mice dosed with vehicle or 20 mg/kg ATRA. B-actin is shown as a loading control. (**B** to **E**) Western blots of co-immunoprecipitation assays to determine the linkage of polyubiquitin chains to cytoplasmic RXRα. HCT-116^Sphk2^ or HCT-116 were exposed to ATRA (5 μM) with or without MG132 (2 μM) for 24 h. Cell lysates were immunoprecipitated with anti-RXRα and then were probed with anti-K48 or -K63 antibodies. K48-linked **(C)** and K63-linked **(D)** ubiquitinated RXRα increased in the immunoprecipitates from HCT-116^Sphk2^ cells as compared to HCT-116 cells (**B** and **D**, respectively). **(F)** Western blots of TRAF2 and TRAF6 expression in HCT-116 and HCT-116^Sphk2^ xenografts from mice dosed with vehicle or ATRA.(**G** and **H**) Western blots of co-immunoprecipitates of TRAF2 and TRAF6 with cytoplasmic RXRα. HCT-116 cells **(G)** or HCT-116^Sphk2^ cells **(H)** were exposed to ATRA (5 μM) with or without MG132 (2 μM) for 24 h. Cell lysates were immunoprecipitated with anti-RXRα and probed with antibodies to TRAF2 and TRAF6. Images of the immunoprecipitates were captured on a FluorChem FC3 image analyzer.

We performed a co-immunoprecipitation assay to investigate the mechanism of SphK2-mediated RXRα ubiquitination. Lysine residues at positions 48 (K48) and 63 (K63) of the ubiquitin polypeptide provide the sites for isopeptide linkages of other ubiquitin molecules. K48-linked polyubiquitin chains act as a signal for the proteasomal degradation of modified substrates while K63-linked polyubiquitin chains produce nonproteolytic signals and provide a scaffold for the assembly of protein kinase complexes [19, 20]. Western blotting analysis showed increased K48 and K63 expression in HCT-116^Sphk2^ xenografted nude mice dosed with 20 and 30 mg/kg of ATRA. As shown in Figure 5A, the levels of K48 expression were strongly increased by 69.7% and 87.6% (p < 0.01 *vs*. vehicle control), respectively; and the levels of K63 were significantly increased by 39.7% and 50.1% (p < 0.05 *vs*. vehicle control), respectively. In contrast, significant changes of K48 and K63 were not observed in HCT-116 xenograft dosed with ATRA (p > 0.05 *vs*. vehicle control). To determine the linkage between the polyubiquitin chains that are conjugated with RXRα, HCT-116^Sphk2^ or HCT-116 cells were exposed to ATRA (5 μM) with or without MG132 (2 μM) for 24 h. Cell lysates were immunoprecipitated with anti-RXRα antibody and then probed with anti-K48 or -K63 antibodies. Ubiquitinated RXRα coimmunoprecipitated with both K48 ubiquitin and K63 ubiquitin. HCT-116 cells demonstrated a weak band of ubiquitinated RXRα expressing K48 ubiquitin (Figure 5B). When HCT-116 cells were exposed to ATRA alone or in combination with MG132, the coimmunoprecipated K48-ubiquitinated RXRα was slightly increased (Figure 5B). In contrast, HCT-116^Sphk2^ cells demonstrated a strong K48-ubiquitinated RXRα coimmunoprecipitate that was further increased when HCT-116^Sphk2^ cells were exposed to ATRA (Figure 5C). These results indicate that overexpression of SphK2 enhances the ATRA-induced RXRα ubiquitination through activation of K48- based poly-ubiquitination. Similarly, HCT-116^Sphk2^ cells demonstrated a much stronger K63-ubiquitinated RXRα coimmunoprecipitate than did HCT-116 cells (Figure 5D and 5E). The coimmunoprecipitate was further increased when HCT-116^Sphk2^ cells were exposed to ATRA (Figure 5E). These data indicate that SphK2 also enhances the ATRA-induced RXRα ubiquitination through activation of the K63- based poly-ubiquitination pathway.

We investigated whether the E3 ubiquitin ligases TRAF2 and TRAF6 were associated with ubiquitinated RXRα. The HCT-116^Sphk2^ xenograft demonstrated higher levels of TRAF2 and TRAF6 expression than those of the HCT-116 xenograft. Further, both TRAF2 and TRAF6 were significantly activated in HCT-116^Sphk2^ xenograft when dosed with ATRA (20 and 30 mg/kg) (Figure 5F). Therefore we investigated whether the ubiquitinated RXRα binds to TRAF2 or TRAF6. Cell lysates were immunoprecipitated with anti-RXRα and probed with antibodies to TRAF2 or TRAF6 by western blotting analysis. TRAF2 and TRAF6 both coimmunoprecipitated in the RXRα protein complex, suggesting that cytoplasmic RXRα binds to TRAF2 and TRAF6. As shown in Figure 5G and 5H, the immunoprecipitates were stronger in HCT-116^Sphk2^ than HCT-116 cells. Importantly, the immunoprecipitates of both TRAF2 and TRAF6 were further stronger in HCT-116^Sphk2^ cells exposed to ATRA (5 μM). These results indicate that TRAF2 and TRAF6 are both involved in the SphK2-enhanced RXRα ubiquitination process. It is likely that TRAF2 and TRAF6 bind with RXRα to form a complex of TRAF2-RXRα or TRAF6-RXRα in the ubiquitin-proteasome system (Figure 5H). When HCT-116^Sphk2^ cells were exposed to ATRA (5 μM) plus MG132 (2 μM), the immunoprecipitate of TRAF6 was even further increased, whereas the immunoprecipitate of TRAF2 was obvious lowered as compared to cells exposure to ATRA alone (Figure 5H). These observations indicate that SphK2-indced RXRα was degraded, suggesting the diverse signaling pathways in SphK2-indced RXRα degradation in polyubiquitination system.

### SphK2 enhances the K63-linked ubiquitin-dependent autophagic degradation of RXRα

**Figure 6:**
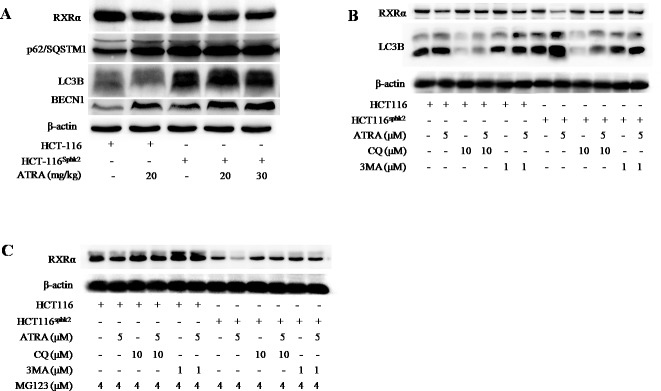
SphK2 enhances the K63-linked ubiquitin-dependent autophagic degradation of cytoplasmic RXRα **(A)** Western blots of LC3B, Beclin-1, p62/SQSTM1 and RXRα in HCT-116 and HCT-116^Sphk2^ xenografts in mice dosed with vehicle or ATRA. **(B)** Western blots of LC3B and RXRα expression in HCT-116 and HCT-116^Sphk2^ cells exposed to ATRA plus chloroquine (CQ) (10 μM) or 3-methyladenine (3MA) (1 μM) for 24 h. **(C)** Western blots of RXRα expression in HCT-116 and HCT-116^Sphk2^ cells exposed to MG132 (4 μM) to block the K48- based proteasomal degradation of cytoplasmic RXRα, in the presence of CQ (10 μM) or 3MA (1 μM) for 24 h.

Since RXRα is conjugated with K63-linked ubiquitin chains, this linkage may provide a scaffold for the assembly of a protein kinase complex leading to the autophagic degradation of RXRα [21, 22]. Western blotting analysis showed higher levels of the autophagy proteins light chain 3 (LC3) and Beclin-1 as well as an increase of p62/SQSTM1 in HCT-116^Sphk2^ xenografts upon ATRA treatment (Figure 6A). The increase in expression levels of these autophagy proteins and p62/SQSTM1 are coincident with decreased RXRα expression, implying that the autophagic signal pathway was involved in the process of RXRα degradation. Further, when HCT-116^Sphk2^ or HCT-116 cells were exposed to ATRA plus chloroquine (CQ) (10 μM), an autophagy inhibitor that blocks autophagosome-lysosome fusion [23], or 3-methyladenine (3MA) (1 μM), an LC3 inhibitor [24], for 24 h, the ATRA-induced degradation of RXRα was blocked (Figure 6B). As shown in Figure 6B, the ATRA-induced degradation of RXRα blocked by CQ or 3MA which resulted in an increase in RXRα expression that was more pronounced in HCT-116^Sphk2^ cells than in HCT-116 cells. At the same time, the level of LC3B was concurrently decreased in HCT-116^Sphk2^ cells exposed to CQ or 3MA (Figure 6B). Using MG132 (4 μM) to block the K48-based proteasomal degradation of RXRα, HCT-116^Sphk2^ cells were again exposed to CQ (10 μM) or 3MA (1 μM) for 24 h and then the level of RXRα was determined. The ATRA-induced degradation of RXRα was obviously blocked after treatment with CQ or 3MA (Figure 6C). These phenomena are more obvious in HCT-116^Sphk2^ cells than in HCT-116 cells. These results indicate that SphK2 mediates ATRA-induced autophagic degradation of RXRα through both a K63-linked ubiquitin-dependent mechanism and K48-linked proteasomal degradation complex system.

## DISCUSSION

In this study, we describe an independent mechanism of ATRA therapy resistance in which Sphk2 enhances a rapid degradation of cytoplasmic RXRα. HCT-116 cells transfected with SphK2 (HCT-116^Sphk2^ cells) demonstrate characteristics of resistance to ATRA therapy as evaluated by *in vitro* and *in vivo* assays. Nude mice xenografted with HCT-116^Sphk2^ and dosed with ATRA at 20 and 30 mg/kg showed markedly less inhibition of tumor growth as compared to nude mice xenografted with HCT-116 cells. To investigate the mechanisms of SphK2-mediated ATRA resistance, we first performed immunofluorescence microscopy to determine the spatial distribution of SphK2 and RXRα in HCT-116^Sphk2^ cells. We found that the transfected SphK2 mainly resided in the nuclei of cancer cells. It has been suggested that the translocation of RXRα from the nucleus to the cytoplasm underlies a unique pathway in the inhibition of growth of various cancer cells [6]. We further analyzed the spatial distribution of RXRα over time in HCT-116 and HCT-116^Sphk2^ cells. In HCT-116 cells, nuclear RXRα is exported to the cytoplasm, leading to an apoptotic effect and cancer growth inhibition. However, in SphK2-transfected HCT-116^Sphk2^ cells, we observed rapid ATRA-induced degradation of RXRα in the cytoplasm. In HCT-116 cells, nuclear RXRα was exported beginning at 2 h post ATRA and most of the exported RXRα remained in the cytoplasm for 24 h. However, in HCT-116^Sphk2^ cells, cytoplasmic RXRα was rapidly degraded from 6 h post ATRA, and most of it had disappeared within 12 h post ATRA exposure. We thus suggest that SphK2-induced degradation of RXRα is linked to resistance of cancer cells to ATRA therapy.

RXRα is required for biological functions of ATRA through the formation of RXRα/RARβ heterodimers. However, ATRA could induce the degradation of RARβ and RXRα in HCT-116^Sphk2^ cells. Our previous report revealed that overexpression of SphK2 mediates ATRA-induced RARβ degradation through an acetylation degradation pathway [16]. Strikingly, in HCT-116^Sphk2^ cells, nuclear RXRα was obviously exported and then was degraded in the cytoplasm upon ATRA treatment. Although some groups have reported that RXRα is also induced by ATRA, it is generally accepted that the natural ligand for RXRα is mainly 9-cis-RA as opposed to ATRA. Since ATRA preferentially induces RARβ expression [25], this raised the question of why RXRα was degraded in HCT-116^Sphk2^ cells? This result prompted us to investigate the fate of RXRα in HCT-116^Sphk2^ cells. It has been suggested that the ratio of RXRα to RARβ is likely one of the key parameters in determining the outcome of retinoid therapy [3]. In response to ATRA, RARβ and RXRα can dimerize to form a heterodimeric nuclear receptor complex that functions as a transcription factor. In HCT-116^Sphk2^ cells, because of ATRA-induced RARβ degradation, we thus suggest that the RARβ/RXRα heterodimer is no longer formed due to loss of RARβ. Under these conditions, the remaining cytoplasmic RXRα induced by ATRA must be degraded for a dynamic balance of RXRα and RARβ in HCT-116^Sphk2^ cells.

Ubiquitination is known for its role in targeting protein aggregates for degradation [26, 27]. In this study, we suggest that SphK2 might enhance the ligand-induced degradation of RXRα through the ubiquitination pathway. We show that cytoplasmic RXRα is more rapidly ubiquitinated in HCT-116^Sphk2^ cells than that in HCT-116 cells. Furthermore, cytoplasmic RXRα is conjugated with K48-linked polyubiquitin chains, which primarily function to target proteins for proteosomal degradation. Since the inhibition of proteosomal activity increases total RXRα protein levels, we suggest that the K48-linked ubiquitination of RXRα functions to target RXRα for proteosomal degradation by the polyubiquitin-proteosome pathway.

However, the K48-linked ubiquitination does not completely degrade the cytoplasmic RXRα probably due to its limited capacity of proteasome [28]. We found that SphK2 might also recruit the K63-linked polyubiquitin chains to cytoplasmic RXRα, therefore initiating the autophagic degradation pathway. Unlike K48-linked ubiquitination, the K63-linked polyubiquitin chain is considered as a regulatory signal that provides a scaffold for the assembly of protein kinase complexes and thus initiates the autophagic clearance of protein aggregates [19, 29]. In the assembly of protein kinase complexes, TRAFs (TNF receptor) are the adaptor proteins that target protein aggregates and facilitate the activation of multiple downstream effectors [29]. TRAF6 and TRAF2 function as ubiquitin ligases that bind with the K63-linked polyubiquitin chains to activate the protein kinase [30, 31]. In this study, HCT-116^Sphk2^ cells demonstrate higher expression of TRAF2 and TRAF6 than HCT-116 cells, suggesting that SphK2 activates the biological activities of TRAF2 and TRAF6 in the recruitment of downstream protein aggregates. Our co-immunoprecipitation analysis showed that TRAF2 and TRAF6 bind more RXRα protein in HCT-116^Sphk2^ cells than in HCT-116 cells. We thus propose that activated TRAF2 and TRAF6 induced by SphK2 may recruit cytoplasmic RXRα to the assembly of protein kinase complexes where it is ubiquitinated by an E3 ligase. Our data indicates that p62/SQSTM1 may also be involved in the recruitment of the K63-linked polyubiquitinated RXRα to the autophagic machinery. p62/SQSTM1 is able to interact directly with ubiquitin and autophagosome protein light chain 3 (LC3) which provides a link between the ubiquitinated inclusions and the autophagy machinery, thereby facilitating the clearance of aggregated proteins from the cells [32, 33]. Our data reveal higher expression levels of p62/SQSTM1 in HCT-116^Sphk2^ cells than in HCT-116 cells. Strikingly, the level of p62/SQSTM1 was significantly increased upon ATRA treatment. We also show that the increase of p62/SQSTM1 is coincident with an increase in autophagy components LC3, Beclin-1, as well as TRAF2 and TRAF6 and UbK48/Ubk63 [34, 35]. These observations are consistent with many other reports that high expression of p62/SQSTM1 recruits LC3 to the autophagosomes though Beclin-1, which provides a link between the ubiquitinated inclusions and the autophagic machinery, thereby facilitating the clearance of the aggregated cytoplasmic RXRα from cancer cells.

Our study provides compelling evidence to support diverse roles for Sphk2 in colonic adenocarcinoma cells resistance to ATRA therapy. We show the complex nature of two independent mechanisms by which Sphk2 enhances a rapid degradation of cytoplasmic RXRα through the ubiquitination pathway: (1) K48-linked proteosomal degradation and (2) K63-linked ubiquitin-dependent autophagic degradation. These results provide comprehensive insights into what Sphk2 functions and how Sphk2 mediates the lack of responsiveness in the retinoids therapy. Our findings will help us to develop strategies that improve the efficacy of retinoid therapy.

## MATERIALS AND METHODS

### Cell lines, cell culture and transfection with SphK2

The human colonic adenocarcinoma HCT-116 cell line was maintained in RPMI-1640 supplemented with 10% (v/v) fetal bovine serum, penicillin–streptomycin (100 IU/ml-100 μg/ml), 2 mM glutamine, and 10 mM Hepes buffer (pH 7.0) at 37°C in a humid atmosphere (5% CO_2_-95% air). Cells were harvested by brief incubation in 0.02% EDTA-PBS (Sigma-Aldrich). The day before transfection, 3 × 10^5^ cells were seeded in a 96-well plate with 100 μL culture medium, to ensure a cell density of 40-60% in each well at the time of transfection. HCT-116 cells were transfected with plasmids pWZL-Neo-Myr-Flag-SphK2 or pWZL-Neo-Myr-Flag-DEST empty vector by mixing with Lipofectamine™2000 (Invitrogen) reagent according to the manufacturer's instructions. Fresh culture medium was substituted 24 h later. Cells were then cultured in RPMI-1640 containing 500 μg/ml of G418 (Gibco). Growth of G418-resistant cells was observed after 7 to 10 days. The selected clones were further grown in medium containing 200 μg/ml of G418 for 4 weeks. G418-resistant clones named HCT-116^Sphk2^ cells were propagated in medium containing 10% FBS in the absence of G418. The expression of SphK2 in HCT-116^Sphk2^ cells was determined by western blotting using primary anti-Sphk2 antibody (ab37977, Abcam) and fluorescence microscopy using primary anti-SphK2 antibody (sc-22704, Santa Cruz). Chicken anti-goat IgG Alexa Fluor® 594 was used as secondary antibody (A-21468, Invitrogen). Images were captured by digital sight camera (Nikon, E 200, ACT-1 software).

### Soft agar colony forming assay

The assay was performed in 35-mm dishes. HCT-116 and HCT-116^Sphk2^ cells (300/dish) were cultured in a cell agar layer containing 10% FBS-RPMI and 0.4% agar (Sigma-Aldrich) on a base agar layer containing 10% FBS-RPMI and 0.6% agar under conditions with or without increasing concentrations of ATRA. HCT-116 or HCT-116^Sphk2^ cells were grown at 37°C for 2 consecutive weeks, and colonies were observed under a light microscope (Nikon SMZ 1500) at 4× magnification.

### Xenografts in nude mice

Animal experiments were approved by the Animal Welfare Committee of Capital Medical University (AEEI-2014-101). Balb/c athymic (nu+/nu+) mice, 6 weeks of age, were purchased from Charles River Laboratories (Beijing, China). Tumors were produced by inoculating the mice with HCT-116 or HCT-116^Sphk2^ cells (1 × 10^7^ per mouse) subcutaneously into the armpit of a mouse. Three weeks later, the rapidly proliferating HCT-116 or HCT-116^Sphk2^ tumor tissue was cut into 1.5 mm thick pieces and inoculated subcutaneously into the armpit of each mouse with a puncture needle. When tumor volumes reached approximately 100 mm^3^, mice were randomly divided into a vehicle control group (n = 6, 0.2 ml olive oil by gavage) and ATRA treatment groups (n = 6, 20 and 30 mg/kg ATRA in 0.2 ml olive oil). All administrations were performed five times per week for three consecutive weeks. Mice were observed daily for any clinical symptoms. Tumor volume and body weight were measured every 3 days. Volume was calculated using the formula, 1/2 × L × W^2^, where length (L) and width (W) were determined in mm [36]. Tumor growth was defined as a ratio to the tumor weight of vehicle controls. Specimens of HCT-116 or HCT-116^Sphk2^ xenografts were removed for further analysis.

### Protein extraction and western blotting analysis

Protein extraction was performed in cultured cells and HCT-116 or HCT-116^Sphk2^ xenografted nude mice. The cultured cells were lysed and protein was extracted using standard methods. In HCT-116 or HCT-116^Sphk2^ xenografts, tumors were homogenized and protein was extracted as described previously [37]. Protein concentration was determined with a BCA kit (Thermo Fisher Scientific). Western blotting assays were performed to determine protein expression. The bound antibodies were visualized with an enhanced chemiluminescence reagent and quantified by densitometry using a FluorChem FC3 image analyzer (Molecular Devices). Densitometric analyses of bands were adjusted with β-actin (Sigma-Aldrich) as a loading control. Primary antibodies included Sphk2 (ab37977, Abcam), RXRα (3085), ubiquitin (3933), K48-linkage specific polyubiquitin (8081), K63-linkage specific polyubiquitin (5621), TRAF2 antibody sampler kit (8347), LC3 (3868), Beclin-1 (3738), SQSTM1/p62 (5114, Cell Signaling), RARβ (sc-552, Santa Cruz).

### Immunofluorescence microscopy

Immunofluorescent staining of RXRα and SphK2 was performed as previously described [38]. HCT-116 or HCT-116^Sphk2^ cells (2.5 × 10^4^) were seeded onto 12-mm glass coverslips in 24-well plates. Cells were fixed in 4.5% paraformaldehyde for 15 min, washed in PBS, permeabilized with 1 × Perm/Wash Buffer (BD Biosciences) for 10 min, washed in PBS, blocked for nonspecific antibody reactions by incubation in solution containing 5% BSA for 30 min, and incubated with primary anti-RXRα or anti-SphK2 (sc-22704, Santa Cruz) antibodies. Secondary antibodies included donkey anti-rabbit IgG (Alexa Fluor® 488 conjugate (A-21206)) and labeled chicken anti-goat IgG (Alexa Fluor® 594 (A-21468), Invitrogen)). Coverslips were mounted with Vectashield® antifade mounting medium containing Hoechst 33258 (Sigma-Aldrich). Confocal laser microscopy was performed on a Leica TCS SP8 AOBS apparatus, using excitation spectral laser lines at 488 nm. Image acquisition and processing were conducted using Leica Confocal Software (Leica Microsystems). Cells stained only with fluorochrome-conjugated secondary antibody were used to set up the acquisition parameters. Signals from different fluorescent probes were taken in a sequential scanning mode. Several fields of view (> 200 cells) were analyzed for each labeling condition.

### Reverse transcription and qPCR assays

HCT-116 or HCT-116^Sphk2^ cells were treated with ATRA (5 μM) for 24 h. Cells were harvested and total RNA was extracted with a Trizol RNA extraction kit (Invitrogen). Reverse transcription was performed with the First Strand cDNA Synthesis kit (Toyobo, Japan). qPCRs assays were performed with the QuantiTect SYBR Green PCR kit (QIAGEN) in a LightCycler 480 apparatus (Roche). Real-time PCR reactions were performed in an ABI 7500 Fast Real-Time PCR System (Applied Biosystems). The primer sequences were: RXRα forward 5′- CTTTGACAGGGTGCTAACAGAGC-3′, reverse 5′-ACGCTTCTAGTGACGCATACACC-3′) [39]. β-actin forward 5′-GTCACCAACTGGGACGACA-3′, reverse 5′-TGGCCATCTCTTGCTCGAA-3′. RARβ forward 5′-GGAACGCATTCGGAAGGCTT-3′, reverse 5′-GGAAGACGGACTCGCAGTGT-3′; β-actin forward 5′-GTGAAGGTCGGTGTCAACGGATTT-3′, reverse 5′-CACAGTCTTCTGAGTGGCAGTGAT-3′ [40].

### Co-immunoprecipitation analysis

HCT-116 or HCT-116^Sphk2^ cells were lysed in RIPA buffer [150 mM NaCl, 5 mM EDTA, 50 mM Hepes (pH 7.0), 0.5% (w/v) sodium deoxycholate, 1% Nonidet P-40 (NP-40), and 10 mM 2-mercaptoethanol] containing 2 mM PMSF, 50 mg/mL of aprotinin A, 25 mg/mL of leupeptin, and 25 mg/mL of pepstatin, for 15 min at 4°C. Cell lysates were then cleared by centrifugation at 12000 × g for 5 min and kept cold on ice. Aliquots of 500 μL of lysate were incubated with 2 μg of specific antibodies. Immunoprecipitates were resolved by 12.5% SDS-PAGE, and the proteins were transferred to polyvinylidine fluoride membranes (Millipore). Membranes were incubated in blocking buffer [1% (w/v) BSA, 5% (w/v) non-fat dry milk, and 0.1% (v/v) Tween-20 in TBS (pH 7.0)] overnight at room temperature. Membranes were subsequently probed with the corresponding antibody in blocking buffer for 16 h: anti-K48-linkage specific polyubquitin antibody (1:1000), anti-K63-linkage specific polyubquitin antibody (1:1000), anti-TRAF2 antibody (1:1000) and anti-TRAF6 antibody (1:1000). Membranes were washed three times with TBS buffer for 5 min and incubated with a 1:5000 dilution of HRP-conjugated anti-rabbit IgG for 1 h at room temperature. After three washes with TBS buffer, the antibody-reactive proteins were detected using a western chemiluminescent HRP substrate (ECL) (Millipore). Immunoprecipitates were visualized with a FluorChem FC3 image analyzer (Molecular Devices).

### Statistical analysis

All experiments were performed at least three times with triplicate samples. Data are presented as means ± SD. Significant differences between groups were determined using either Wilcoxon scores or the Kruskal–Wallis test. A p -value of 0.05 was considered significant.
